# Synthesis of BaTiO_3_ nanoparticles as shape modified filler for high dielectric constant ceramic–polymer composite†

**DOI:** 10.1039/d0ra04196c

**Published:** 2020-08-07

**Authors:** Taehee Kim, Hanwhuy Lim, Youngkwan Lee, Baek-Jin Kim

**Affiliations:** Korea Institute of Industrial Technology (KITECH) Cheonan South Korea bjkim@kitech.re.kr; Department of Chemical Engineering, Sungkyunkwan University Suwon South Korea; Department of Chemical and Biomolecular Engineering, Yonsei University Seoul South Korea

## Abstract

Coral-like structured barium titanate (BaTiO_3_) nanoparticles were synthesized as filler for a high dielectric elastomer. The nanoparticle size, and shape, and the reactivity of the synthesis were modified according to temperature, time, pH, and precursor materials. Dielectric properties of poly(dimethylsiloxane) (PDMS) composites were estimated by volume fractions of BaTiO_3_ of 5, 10, and 15 vol% for both sphere and coral-like shapes. As a result, coral-like BaTiO_3_–PDMS composites had the highest dielectric constant of 10.97, which was 64% higher than the spherical BaTiO_3_–PDMS composites for the 15 vol% fraction. Furthermore, the phase transition process and surface modification were applied to increase the dielectric properties through calcination and improved particle dispersion in the elastomer using polyvinylpyrrolidone (PVP). The dispersion of the PVP coated BaTiO_3_–PDMS composite was improved compared to pristine BaTiO_3_ as shown by SEM imaging. The coral-like BaTiO_3_ embedded composite could be used for electronic devices such as piezoelectric devices or electro-adhesive grippers, which require flexible and high dielectric materials.

## Abstract 2

Coral-like structured barium titanate (BaTiO_3_) nanoparticles were synthesized as filler for a high dielectric elastomer.

## Graphical abstract

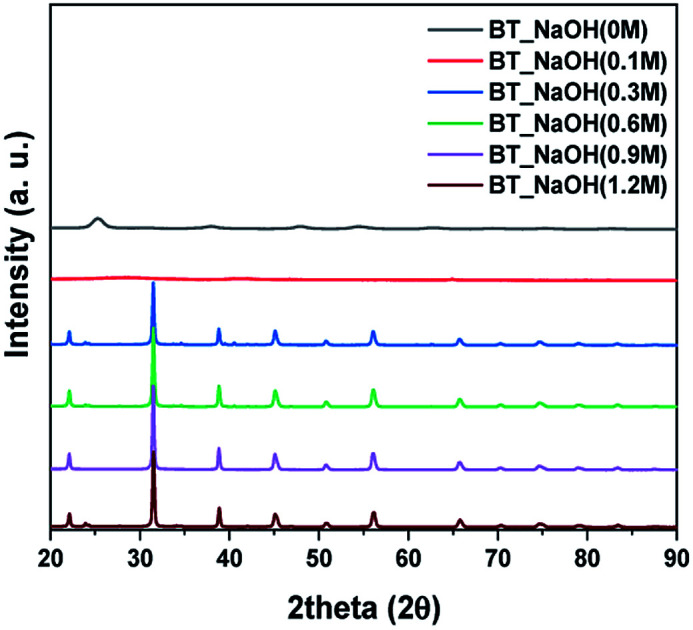

## 1. Introduction

Robotic arm grippers use various energy sources including motors, electrical power, and vacuum adsorption. However, there is a limitation in the application on flat surfaces or atypical objects.^1^ On the other hand, the use of adhesive force could allow atypical objects and flat films to be lifted.^2^ An electro-adhesive force occurs when a voltage is applied to an electrode pattern, which forms an interdigitated pattern, generating an electric force to lift an object.^3^ Basically the force of electro-adhesion was calculated though the following equations.
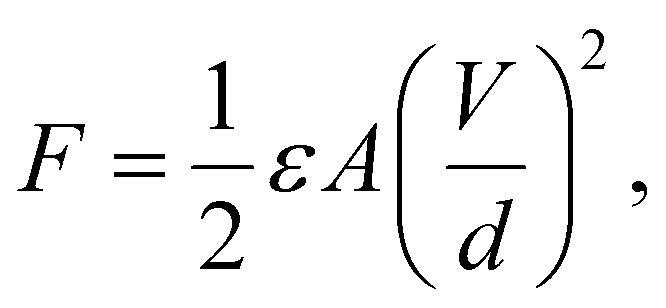
where *ε* is the dielectric permittivity of insulated layer, *A* is the area of the electrode, and *V*, *d* are the applied voltage difference and the equivalent gap between the electrodes. These parameters are related to each other. For example, the limitation of applied voltage (*V*) are depending on *d* and *ε*, and *A* and *d* are inversely related to each other within a certain area. Most of recent studies about electro-adhesive gripper were mainly related to *A*, *V*, and *d* except *ε*.^1–5^ To increase dielectric permittivity, many people use dielectric materials such as TiO_2_, ZrO_2_, and BaTiO_3_, amongst others.^6^ BaTiO_3_ (BT) is of particular interest due to its perovskite structure and high dielectric constant of more than 1000, which is significantly higher than other ceramics, for example, 3.9 for SiO_2_.^7,8^ BT is used in fields requiring high piezoelectricity and high dielectric constants because of its polarization inversion and high dielectric constant.^9^ However, the material for the insulating layer needs to be flexible and stretchable in order to grab the various shaped objects. In many studies of high dielectric constant composites, polyvinylidene fluoride (PVDF) with a relatively high dielectric constant has been used among polymer materials,^10,11^ however, it is not suitable to apply on grippers used for atypical objects because of its poor elasticity. PDMS is one of the suitable polymers which has a low elastic modulus *E* of approximately 0.002 Gpa and is easy to fabricate as a composite.^12^ Therefore, in this research, BT nanoparticles (NPs) and PDMS were compounded as a material for an insulating layer on an electric pattern. Moreover, when BT is formed as a tetragonal structure, spontaneous polarization is formed along the *c*-axis. It has been shown that this structure has a higher dielectric constant than a cubic structure where spontaneous polarization does not appear.^13^ One of the methods to obtain a phase transition is *via* calcination which changes the lattice structure by applying high heat over a critical point.^14^ Calcination is a heat treatment process in which the phase transition is obtained by heating below the melting point. In order to have a high dielectric constant, this study aimed to increase the dielectric constant by phase transition of BT from the cubic to the tetragonal structure. However, when compounding BT and PDMS, the two materials do not mix uniformly and NPs sink or agglomerate due to low interfacial adhesion between NPs and PDMS.^15,16^ To solve this problem, NPs were modified by polyvinylpyrrolidone (PVP) which is often used as a stabilizer.^17–20^ BT was synthesized by the hydrothermal method, which allows for synthesis in various particle forms, for example, spherical shape, coral-like shape and rod-like shape.^21–23^ In particular, spherical BT have been used in many studies for composites with high dielectric constants, and in the case of rod-like shape BT, many studies have investigated the aspect ratio which affects the dielectric constant.^24,25^ On the other hand, few studies have investigated composites of BT in the coral-like form,^26^ and the dielectric properties of BT dispersed in PDMS elastomer have rarely been reported. A coral-like morphology is expected to have a larger surface area than spherical NPs, and the shape is expected to affect the reaction with the polymer matrix, the dielectric properties, and the dispersion. Therefore, in this study, BT were synthesized with a coral-like shape, and a phase transition and surface modification was used to improve the properties of BT–PDMS composites for use as dielectric elastomers. Previous research used spherical and rod-shape NPs as dielectric composite fillers, but this study focused on newly shaped filler, coral-like BT, which were rarely studied in the dielectric field. The coral-like BT composite showed better dielectric properties than the spherical BT composite and it could be considered as the effect of large surface area. It is important to have high dielectric properties and stretchability of dielectric composite for the applications such as soft-robotic gripper for lifting atypical objects. Thus, the PDMS elastomer was used instead of PVDF which was widely used as a dielectric matrix. In order to solve the NPs aggregation and agglomeration in polymer, which can be a fatal defect in the ceramic–polymer composite, a chemical treatment of surface modification was performed to obtain a composite in which NPs were uniformly dispersed in the matrix. The dispersion of NPs and the dielectric constant were measured precisely according to the shape and synthesis conditions of the BT.

## 2. Experimental

### 2.1 Materials

Titanium(iv) butoxide (Ti(C_4_H_9_O)_4_, reagent grade, 97%), barium chloride dihydrate (BaCl_2_·2H_2_O, ACS reagent, ≥99%), barium nitrate (Ba(NO_3_)_2_, ACS reagent, ≥99%), polyvinylpyrrolidone ((C_6_H_9_NO)_*n*_, *M*_w_ = 10 000), acetic acid (CH_3_COOH, glacial, ACS reagent, ≥99.7%) was purchased from Sigma-Aldrich (Saint Louis, USA). Barium hydroxide monohydrate (Ba(OH)_2_·H_2_O, 95%) was purchased from Alfa Aesar (Massachusetts, USA), sodium hydroxide, bead (NaOH, >98.0%), hydrogen peroxide (H_2_O_2_, 34.5%), ethyl alcohol (EtOH, C_2_H_5_OH, 94.5%) and isopropyl alcohol (C_3_H_7_OH, 99.5%) were purchased from Samchun Chemicals (Pyeongtaek-si, South Korea). Poly(dimethylsiloxane) (PDMS, (C_2_H_6_OSi)_*n*_, Sylgard™ 182 Silicone Elastomer) was purchased from Dow Silicones Corporation (Midland, MI USA), barium titanate (Spherical BT, 99.9%, 100 nm, Cubic) was purchased from US Research Nanomaterials, Inc. (Houston, TX USA). Deionized water (DI water) was used.

### 2.2 Synthesis of coral-like BT precursors

Coral-like BT were synthesized by a hydrothermal method. First, DI water (56 ml) was added to a 100 ml volume PPL (poly-*para*-phenol) liner followed by NaOH with concentrations of 0.1 M (0.006 mol, 0.24 g), 0.3 M (0.018 mol, 0.72 g), 0.6 M (0.036 mol, 1.44 g), 0.9 M (0.054 mol, 2.16 g), and 1.2 M (0.072 mol, 2.88 g). When NaOH well dissolved, Ti(C_4_H_9_O)_4_ (0.009 mol, 3.06 ml) was added dropwise and stirred at 1200 rpm for 10 minutes. In order to obtain a molar ratio of Ti : Ba of 1 : 1, Ba(NO_3_)_2_ (0.009 mol, 2.35 g) was added and mixed for additional 20 minutes. Other Ba precursor materials, BaCl_2_·2H_2_O (0.009 mol, 2.20 g) and Ba(OH)_2_·H_2_O (0.009 mol, 1.70 g) were used for other Ba source conditions. After mixing, the PPL liner was placed in the hydrothermal reactor and put into an oven. The oven was set according to the reaction temperature (150–210 °C) and reaction time (6–24 h). After the reaction was completed, the reactor was cooled to room temperature and the solution was washed twice with acetic acid aqueous solution (5 vol%), to remove impurities remaining in the solution, and BaCO_3_ which is a side product during the hydrothermal reaction.^27^ The NPs were then washed with DI water and twice with EtOH. The washed NPs were then dried in a vacuum oven at 60 °C for 24 hours.

### 2.3 Phase transition of BT

Calcination was used as the method for the phase transition of the BT with different shape of NPs, the pristine spherical BT (sBT) and pristine coral-like BT (cBT), respectively. sBT were used by purchasing a cubic structure of 100 nm size. Calcination of pristine BT was carried out in a tube furnace (model TF830, LAB HOUSE) at reaction temperatures 750–950 °C and reaction times 6–15 h in an air atmosphere. Calcination of the cBT was carried out in a N_2_ atmosphere for 9 hours at 950 °C, and slowly cooled to room temperature after the reaction was completed. After cooling the NPs, they were stored in a desiccator to prevent the absorption of moisture from the air.

### 2.4 Hydroxylation of calcined BT

Calcined BT are difficult to react with PVP due to the absence of OH functional groups on the surface which are mostly removed from the surface and lattice during calcination.^28^ Therefore, hydroxylation was performed to form OH groups on the NPs surface. The reaction sequence was carried out in a 3 neck round flask (250 ml) with H_2_O_2_ (aqueous solution, 34.5%) (3.29 mol, 100 ml), BT (0.018 mol, 4.29 g). A sonicator (model Power sonic 410, Hwashin Tech) was used for 30 minutes to disperse the NPs. The dispersed solution was refluxed for 6 hours at 106 °C. After the reaction was completed, the mixture was cooled to room temperature and centrifuged for 5 minutes at 5000 rpm using a centrifuge (model Supra 22K, Hanil Scientific Inc.) to obtain the extracted NPs. The NPs were then washed twice with DI water to remove impurities. Finally, they were dried for 12 hours at 80 °C in a vacuum oven.

### 2.5 Surface modification of BT

**Scheme 1:**
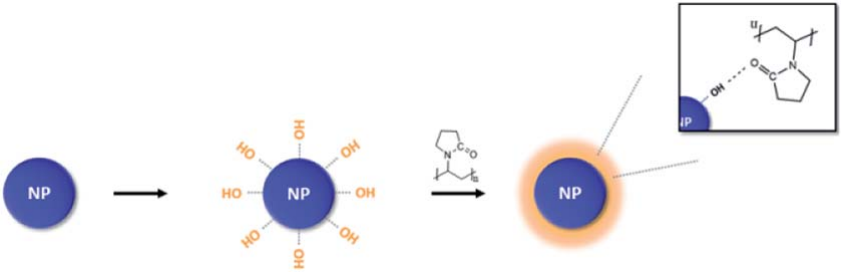
PVP surface modification of BT after calcination.

After OH hydroxylation, the surface of the BT was modified with PVP. The molecular weight of PVP used in modification was 10 000 g per mole (Scheme 1). Calcined BT was modified by PVP with BT : PVP molar ratios of 10 : 0.1, 10 : 0.5, 10 : 1 and 10 : 2 in reaction. The reaction process is as follows. EtOH (99.5 ml) was placed in a round flask (250 ml) and BT (0.005 mol, 1.20 g) were added in a volume ratio of EtOH : BT (500 : 1). The NPs were then dispersed by sonication for 1 hour. Once the NPs were well dispersed in the solvent, the PVP was put into the sonicator at the desired molar ratio and sonicated for 1 hour. After the dispersion was completed, the reaction was performed for 24 hours at 540 rpm using a magnetic bar. The solution was then centrifuged at 5000 rpm for 20 minutes. To remove untreated NPs and impurities, the solution was washed twice with EtOH. After washing, the filtered NPs were dried at 80 °C for 24 hours in a vacuum oven. The dried NPs were slowly crushed with a mortar and pestle to separate the aggregated NPs.

### 2.6 Preparation of BT–PDMS composites

For PDMS, the resin and curing agent were mixed at a recommended ratio of 10 : 1. The procedure for making composites is as follows. First, PDMS resin and BT were mixed with a spatula to separate agglomerated NPs. BT was then mixed with PDMS elastomer until a practicable maximum volume value of 15 vol%. It was difficult to operate at higher than 15 vol% due to the high viscosity. After that, a planetary mixer (model ARE-310, THINKY Corporation) was used for 2 minutes at 2000 rpm to physically disperse the NPs in the PDMS elastomer. This was repeated after it had cooled down. Bubbles were generated by mixing process, and so it was located in a vacuum oven for 1 hour to remove these bubbles before adding the curing agent. After the addition of the curing agent, it was further mixed with a planetary mixer for 2 minutes at 2000 rpm and put into the vacuum oven for another 1 hour. Finally, the sample was poured into a mould, which was prepared to measure the dielectric constant, and the composite was cured in an oven at 80 °C for 1 hour.

### 2.7 Characterization

X-ray diffractometer (XRD, model MiniFlex600, Rigaku) was used to confirm the lattice structures under the conditions of X-ray generator (Cu Kα, 40 kV, 15 mA), step size (0.02°) and step speed (5° min^−1^). A pH meter (model Starter3100, OHAUS) was used as an analyzer for measuring the pH of the solution in the hydrothermal synthesis. Fourier-transform infrared spectroscopy (FT-IR, model Nicolet 6700, Thermo Fisher Scientific) was used to analyse the IR spectrum of the reaction products. To determine the weight loss of PVP on the modified BT surface, the analysis was performed using a Thermogravimetric Analyzer (TGA, model Pyris1 TGA, PerkinElmer). After OH hydroxylation, X-ray photoelectron spectroscopy (XPS, model K-Alpha+, Thermo Fisher Scientific) was used to confirm the elements of the synthesized BT surface. The shape of the NPs and dispersion in PDMS elastomer composites were observed in SEI mode with an acceleration voltage of 5 kV using a Field Emission Scanning Electron Microscope (FE-SEM, model JSM-6701F, JEOL), and a Pt coating was processed for 180 seconds before analysis. Ultramicrotome (model CR-X, RMC) was used on cryo mode to prepare a uniform thin TEM sample under 100 nm thickness prior to TEM image measurement using FE-TEM (model Tecnai G2 F20 X-Twin, FEI). An impedance measurement system (model 4294A, Agilent) was used to evaluate the dielectric properties of the PDMS elastomer composites in a frequency range of 40 Hz to 30 MHz at room temperature. To analyze surface hardness according to the experimental condition, nanoindentation was performed by nanoindenter (model ZHN, Zwick Roell). It was performed under strain = 0.1 s^−1^, maximum depth = 9 μm conditions and the average value was obtained from the average value of 5 different points. To analyze the specific surface area of sBT and cBT, a surface area analyzer (BET, model Autosorb-iQ/MP, Quantachrome Inst.) was used in conditions of pre-treatment at 300 °C for 3 hours in N_2_ gas. The specific surface area was calculated by the Brunauer–Emmett–Teller (BET) equation during the measurement.

## 3. Results and discussion

### 3.1 cBT synthesis by hydrothermal reaction

#### 3.1.1 XRD pattern and FE-SEM morphology analysis

**Fig. 1:**
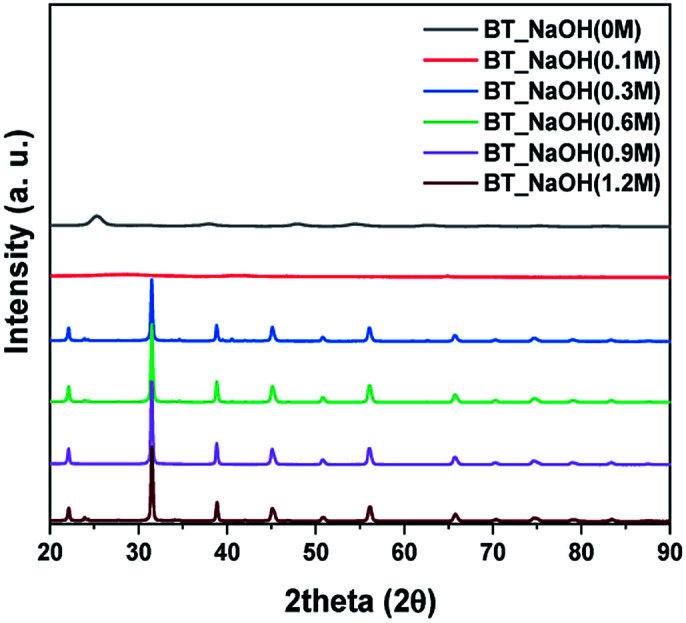
XRD data of synthesized particles by NaOH concentration (0.1 M to 1.2 M).

The X-ray powder diffraction patterns were measured to verify the synthesized BT series. First, the results of hydrothermal synthesis were performed with various NaOH concentrations. The fixed reaction factor was as follows: temperature, time and precursor material such as 150 °C, 15 hours, and barium nitrate (Ba(NO_3_)_2_). As shown In Fig. 1, when 0 M NaOH was used, the resulting XRD pattern showed the TiO_2_ phase (ICDD No. 00-021-1272), and when 0.1 M NaOH was used the XRD pattern showed the BaCO_3_ phase (ICDD No. 00-045-1471). On the other hand, the BaTiO_3_ phase (ICDD No. 01-075-0215) was completely synthesized under 0.3 M NaOH conditions. These results showed that the NaOH concentration has a significant effect on the synthesis. Previous studies have reported that BT can be stably synthesized with strong base above pH 12, but BaCO_3_ or Ba^2+^ ions occur below pH 12.^29^ For comparison, NaOH concentrations of 0.1 M, 0.3 M, 0.6 M, 0.9 M and 1.2 M coincide with pH 12.97, pH 13.16, pH 13.29, pH 13.45 and pH 13.56, respectively. As a result, this indicates that BT was synthesized when pH was over pH 13 at 0.3 M NaOH concentration. This is because the BaCO_3_ phase can be stable up to pH 13.^29^ And in the synthesized BT XRD pattern, the (200) peak for the cubic phase BT was observed at 2*θ* = 43°.

**Fig. 2:**
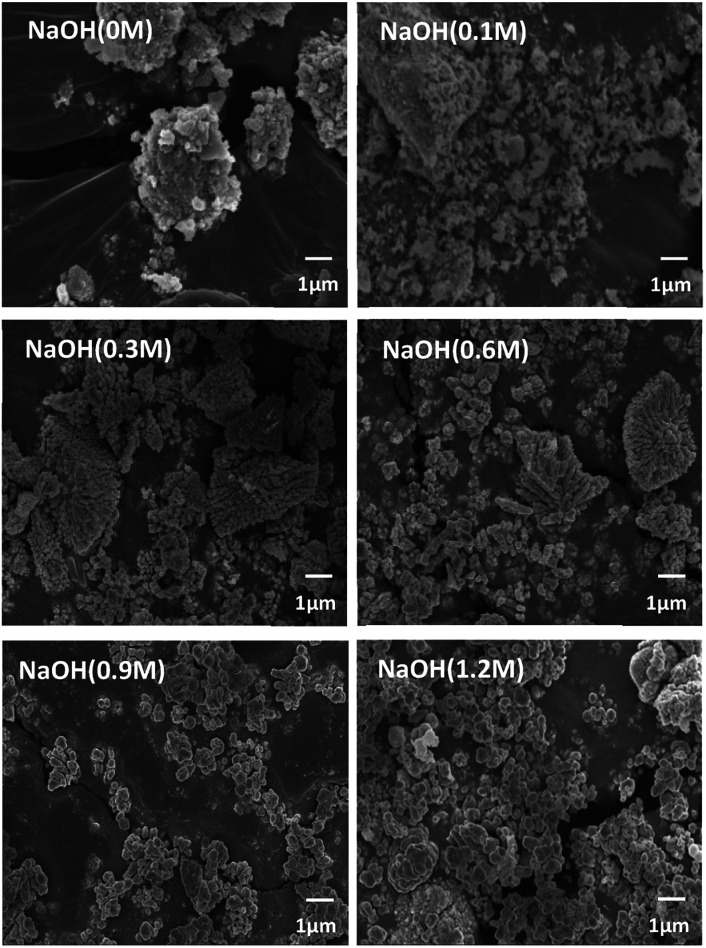
FE-SEM images of particles after hydrothermal synthesis by NaOH concentration condition (×5000).

The morphology of BT was varied according to the NaOH concentration as shown in Fig. 2. In the case of 0 M and 0.1 M NaOH concentrations, the NPs are not clearly observed. However, coral-like NPs were observed at 0.3 M, and both the coral-like NPs and the spherical NPs were observed at 0.6 M. Eventually, only spherical NPs were observed at 0.9 M and 1.2 M. This suggests that the pH value during the reaction determines not only the synthesis of BT but also plays an important role in the shape of the NPs. It is predicted that the nuclei of unstable NPs are bonded to each other as they grow like coral, and as the NaOH concentration increases, the growth is promoted to form spherical stable NPs at each nucleus.^30^ Because coral-like NPs were obtained from 0.3 M NaOH concentration, the coral-like NPs were synthesized at 0.3 M condition.

**Fig. 3:**
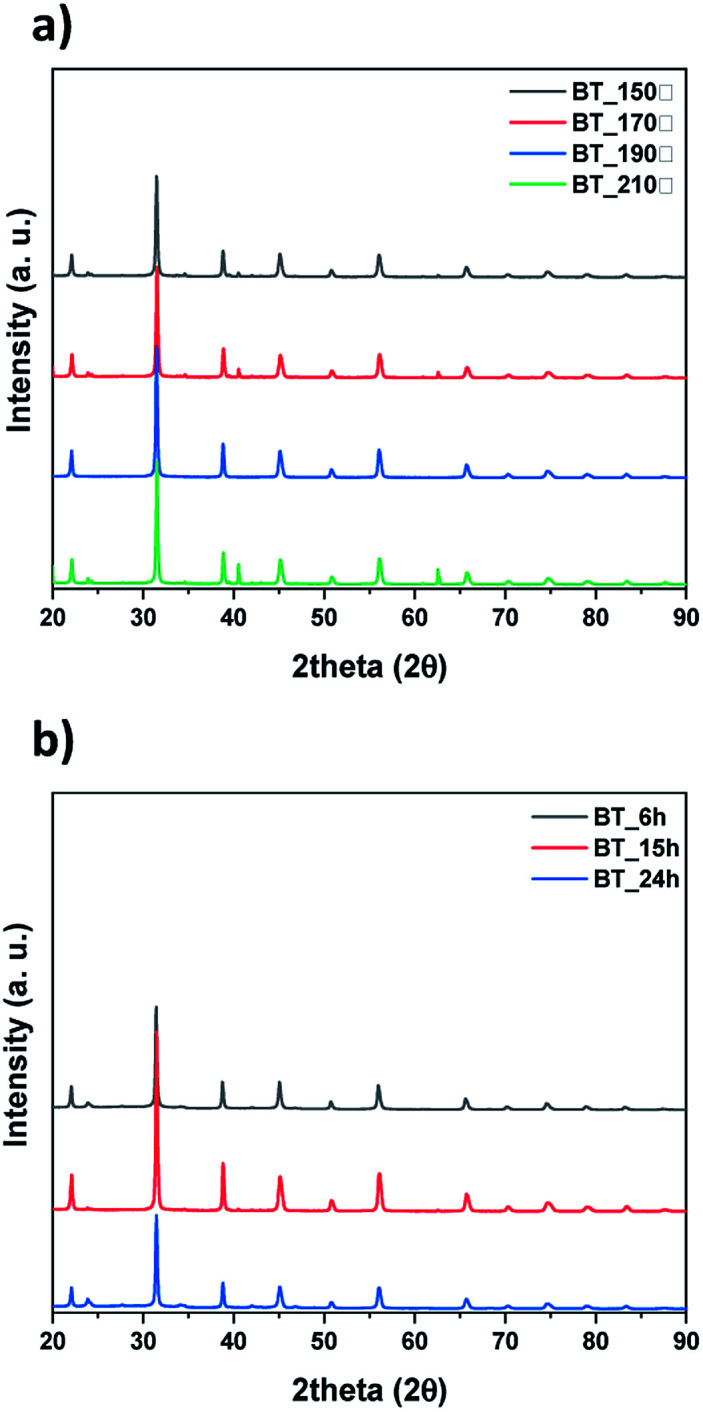
XRD data of synthesized particles by conditions, (a) reaction temperature and (b) reaction time.

**Fig. 4:**
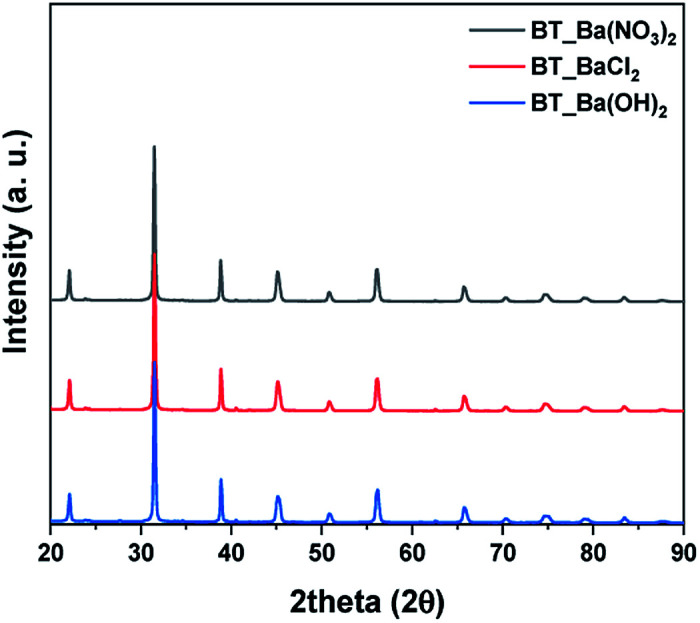
XRD patterns after hydrothermal synthesis by Ba sources condition, Ba(NO_3_)_2_, Ba(OH)_2_ and BaCl_2_.

Next, we observed the difference associated with the reaction temperature. Other conditions were set as follows: a time of 15 hours and barium nitrate (Ba(NO_3_)_2_) as the Ba source. As a result, all the NPs synthesized at 150 °C, 170 °C, 190 °C and 210 °C were identified as BT (ICDD No. 01-075-0212) in the XRD pattern Fig. 3a. Although the BaCO_3_ lattice structure peak still remained at a reaction temperature of 210 °C, it was removed after washing the NPs with 5 vol% acetic acid aqueous solution. The NPs shape was also observed according to reaction temperature by FE-SEM, showing coral-like shapes (Fig. S1†). It showed that NPs are transformed into the coral shape when the reaction temperature is over 190 °C. The reaction time was varied from 6 hours to 24 hours, and the other conditions were fixed as follows: 0.3 M, 190 °C and barium nitrate (Ba(NO_3_)_2_) as the Ba source. And all results were synthesized as BT as shown in Fig. 3b, and the morphology was observed to be coral-like in the FE-SEM images (Fig. S2†). Coral-like NPs were synthesized clearly after 15 hours, and the size of the coral-like NPs grew up 2 times bigger after 24 hours. Finally, the cBT synthesis conditions were optimized at 0.3 M (NaOH concentration), 190 °C and 15 hours. In order to compare the effect of the Ba source, two kinds of Ba sources were investigated; BaCl_2_·2H_2_O and Ba(OH)_2_·H_2_O. In Fig. 4, XRD patterns showed that BT was synthesized in all three starting materials conditions.

**Fig. 5:**
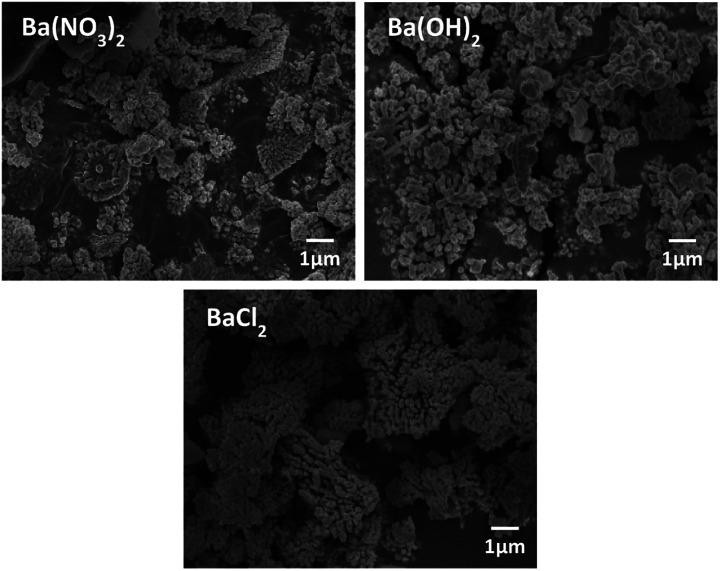
FE-SEM images of particles after hydrothermal synthesis by Ba sources condition, Ba(NO_3_)_2_, Ba(OH)_2_ and BaCl_2_.

The morphology of BT was varied according to the Ba source as shown in Fig. 5. In the case of Ba(OH)_2_·H_2_O, NPs have a mixture of coral-like and spherical shapes. However, for Ba(NO_3_)_2_, coral-like shapes were dominant, and the NPs from the BaCl_2_·2H_2_O conditions as also showed a coral-like shape. This means that the type of anion in the Ba source is an important factor for adjusting the shapes of the NPs. Especially, when the BaCl_2_ source is used as precursor, it has weak interaction with each other. Therefore, anions have only a few active sites around the particles. Lack of active site make BT particles from coral-like shape.^31^ Finally, the BT synthesis for coral-like NPs carried with a NaOH concentration of 0.3 M, reaction temperature 190 °C, reaction time 15 hours and BaCl_2_·2H_2_O as the Ba source.

#### 3.1.2 BET surface area of BT

**Fig. 6:**
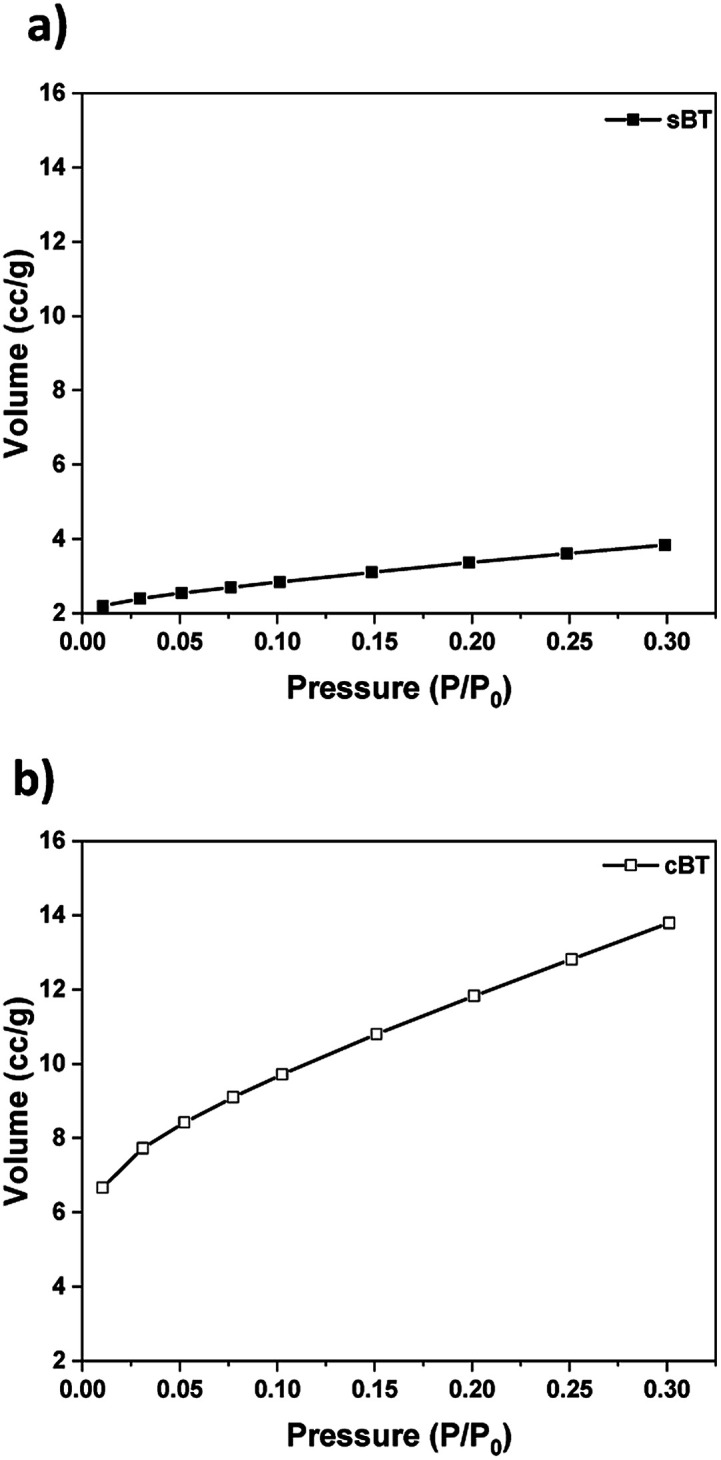
BET specific surface area of particles. (a) sBT and (b) cBT.

The specific surface area of sBT and cBT was measured as shown in Fig. 6. The surface area was measured as 11.84 m^2^ g^−1^ and 42.02 m^2^ g^−1^ for sBT and cBT, respectively. cBT has a larger surface area approximately 4 times than that of sBT indicating that the nuclear growth of coral reef brought high surface area. It is expected that the interaction area between the polymer and the NPs would be wider in the coral-like form. Since the surface of the ceramic NPs is highly activated, cBT NPs become more active and cause highly effective on the dielectric properties.^32^ In addition, the polymer chains interacted around the NPs are layered on the particle surface, and become immobile due to the strong bonding.^33^ Therefore, when cBT mixed with PDMS, the amount of immobilized polymer on the particle surface is increased which allow relatively high *T*_g_ value.

### 3.2 Phase transition of cBT and sBT

#### 3.2.1 XRD pattern and FE-SEM morphology analysis

**Fig. 7:**
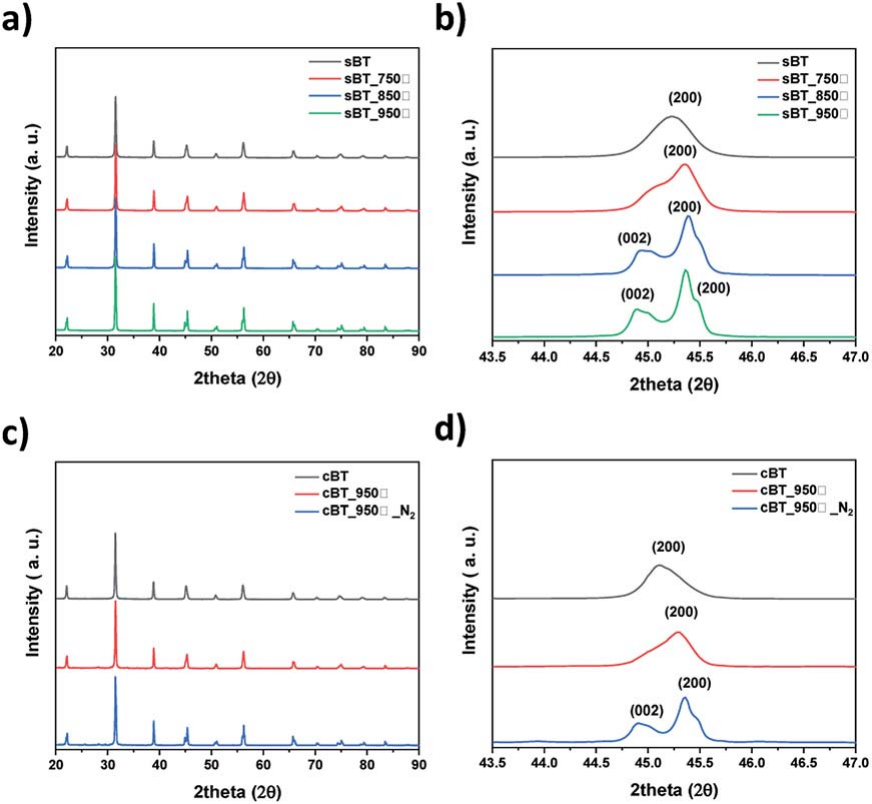
XRD patterns of BT by calcination temperature (a) sBT, (b) sBT in range of 2*θ* = 43.5°–47° and (c) cBT, (d) cBT in range of 2*θ* = 43.5°–47°.

**Fig. 8:**
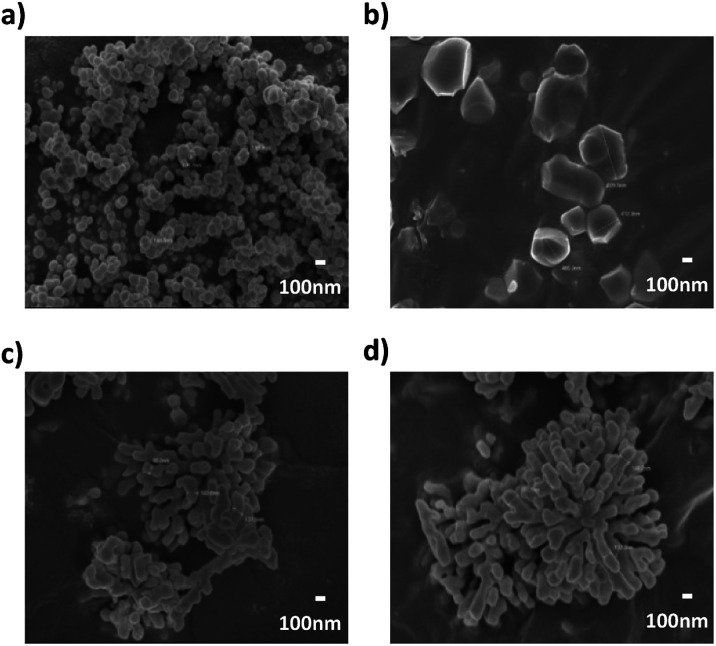
FE-SEM images of BT NPs (×30k) (a) sBT, (b) sBT950, (c) cBT and (d) cBT950.

To enhance the dielectric properties of BT, phase transition of the synthesized BT was carried out using the calcination method and verified through XRD patterns as shown in Fig. 7. sBT were also calcined to compare with cBT at 950 °C in the air (sBT950). Calcination was carried out in a tube furnace and reaction time was fixed as 9 hours. The sBT calcined at 750 °C did not transform to the tetragonal phase, however, sBT calcined over 850 °C showed the tetragonal phase BT (ICDD No. 01-081-2201) as confirmed by observation of (200) and (002) peaks in range 2*θ* = 44°–46° in Fig. 7b. The (200) peak is divided into (200) and (002) peaks when BT transfer from the cubic to the tetragonal phase.^34^ cBT was also calcined at the same conditions as that of sBT. In Fig. 7c, however, it showed that the cBT did not transform from the cubic to the tetragonal phase under the same conditions. Thus, the atmosphere was changed from air to N_2_ to obtain the tetragonal phase. In the XRD peaks (Fig. 7d) both the (200) and the (002) peaks were observed around 2*θ* = 44°–46°. This can be interpreted as meaning that the large surface area of the cBT NPs has excellent reactivity, preventing the removal of OH groups from the NPs even when a small amount of oxygen or H_2_O is present in the air.^35^ The NPs shapes before and after calcination were observed by FE-SEM as shown in Fig. 8. The sBT were grown from 140 nm in the cubic phase to nearly 500 nm when transformed to the tetragonal phase, on average, and the edge of the NPs was also slightly angled. It was confirmed that coalescence occurred as the phase transformed at high temperatures between the interfaces of NPs. In the case of cBT, the thickness of one coral reef was 120 nm on average, and it was only grown to 150 nm after calcination while maintaining the coral-like form.

#### 3.2.2 Dielectric properties after phase transition

**Fig. 9:**
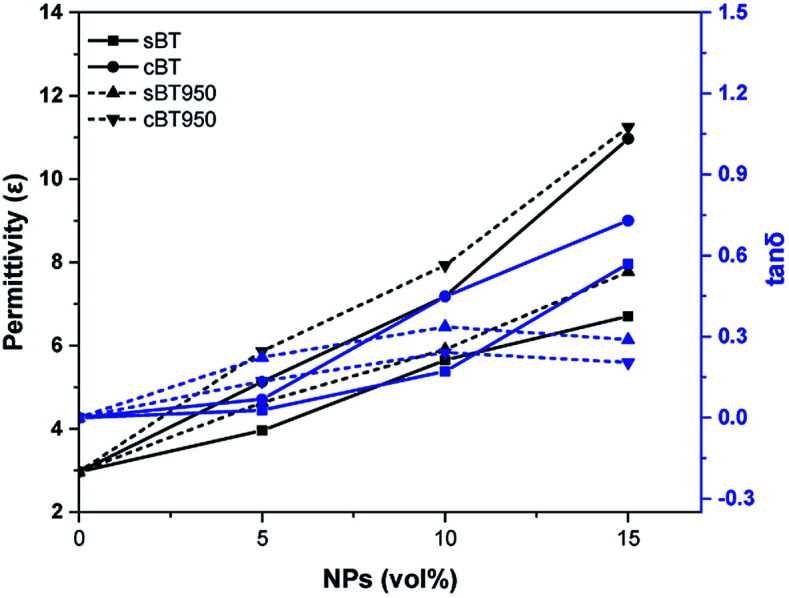
Dielectric properties of BT–PDMS composites before and after calcination. Permittivity (*ε*) and tan *δ* at 0.1 MHz.

The effect of calcination was compared through dielectric property of BT–PDMS composite. It was mixed with a planetary mixer and sampled at 25 mm × 25 mm wide and 2 mm thick for estimated permittivity. The permittivity of BTs and phase transferred BTs were shown in Fig. 9. It was proportioned to the volume percent of BTs in every composite. Furthermore, it showed that cBT–PDMS composites have much higher values of permittivity than sBT–PDMS composites. This was especially notable in the 15 vol% composite, which increased by 64% compared to the sBT–PDMS composite. This is reasonable compared to the result from rod-like BT filler reported dielectric constant enhancement by the higher aspect ratio of BT fiber.^36^ It could be suggested that the branch of cBT increases dielectric constant compared to sBT–PDMS composites. On the other hands, the dielectric loss is proportional to the content of NPs except 15 vol%. The loss of the cBT–PDMS composite showed relatively lower value than the composite of sBT–PDMS. Furthermore, the PVP coated NP have benefit of low dielectric loss for every vol% condition. It indicated that the amount, and the shape of NPs filler affected to the dielectric properties of composite and 15 vol% cBT–PDMS showed the highest dielectric constant with relatively low dielectric loss as shown in Fig. 9.

#### 3.2.3 Surface hardness after phase transition

**Fig. 10:**
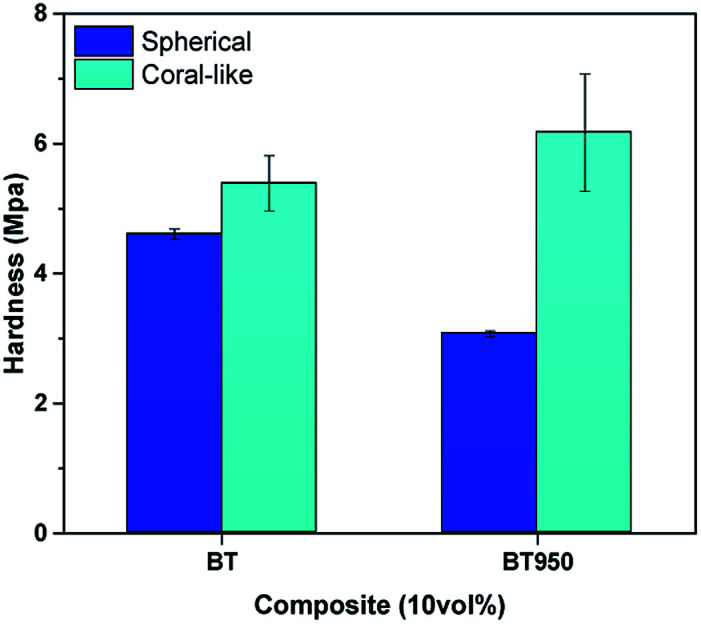
Surface hardness of 10 vol% composite. sBT, sBT950, cBT and cBT950 composites.

In Fig. 10, nanoindentation was examined to measure the surface hardness of 10 vol% composites under strain rate (0.1 s^−1^) and maximum depth (9 μm) to reduce error.^37^ The cBT composite showed a relatively high surface hardness than sBT composite. In addition, in the case of sBT composite, the hardness decreased after phase transition as sBT950. It is expected that the agglomeration of the NPs becomes more severe and sinks after the removal of OH group, so that the NPs are not dispersed well in the upper surface layer resulting in lower surface hardness. On the other hand, the hardness of cBT composite was maintained when cBT transferred to cBT950. Although the cBT950 NPs tried to be settled as the surface OH group removed after the phase transition, the coral reef morphology of cBT950 help it to be dispersed in the matrix, relatively. Finally, the surface hardness of PVP coated experiment was improved over 55% by the effect of mechanical strength of well-dispersed NPs.

### 3.3 Hydroxylation of cBT950 and sBT950

Attaching the OH functional group to NPs surface through hydroxylation was carried out for surface modification of BT950. In order to graft PVP to BT950 surface, OH groups are required on the NPs surface to enable hydrogen bonding with C=O groups of PVP.^38^ Hydroxylation is required because the OH groups in BT were removed during calcination. XPS data of BT950 and hydroxylated BT950 (BT950-OH) is compared in Fig. S3 and S4.† In photoelectron spectra of O 1 s, the O ion corresponds to the binding energy range of 532–531.1 (eV), representing H_2_O and the oxygen ion on the oxide surface, and the peaks appearing at 531.1–530.6 (eV) are OH^−^ ions in alkali and peroxide, and O^2−^ ions of oxide ions appeared in the range of 530.6–527.7 (eV).^39^ The integrated area of the OH^−^ peak was compared after hydroxylation, showing that the area of cBT950 increased 3 times after hydroxylation. In Fig. S4,† the OH^−^ integrated peak area of sBT950 at 530.87 (eV) was increased 3.35 times after hydroxylation (Table S2†). When NPs were chemically treated by H_2_O_2_, OH functional group was generated on Ba ions and it leads NPs to become a Brönsted-basic sites. It help to solve in EtOH and facilitate the PVP surface modification reaction.^40^ The photoelectron spectra of C 1 s demonstrated the peaks for C–C, C–H, C–O, and C=O from the BT. The element ratio of BT was also confirmed by XPS analysis of Ba ions and Ti ions after the synthesis of the coral-like NPs. As a result, the Ba : Ti ratio was measured as 12.08 : 12.45 atomic%, indicating that the synthesis was performed in an almost 1 : 1 ratio. The elemental ratios of O and C ions were measured to be 46.59%, 29.87%, respectively. The O ions of BT measured higher value than stoichiometric ratio due to OH groups and impurities from XPS equipment.

### 3.4 Surface modification of cBT950 and sBT950

The surface modification of NPs was identified by particle size analysis (PSA). The PSA measurement showed that the particle sizes increased after PVP modification as shown in Fig. S5 and S6.† The average particle size of sBT950 increased from 500 nm to 658 nm after PVP modification compared to the cBT950 from 532 nm to 600 nm according to the amount of PVP. When the amount of PVP is low, partially coated PVP acts as a bridge among the NPs. On the other hands, excess PVP covered multiple NPs forming agglomeration NPs.^41^ I order to find the optimal conditions, we processed various range of chemical treatment depending on the NPs : PVP molar ratio as 1 : 0.1, 1 : 0.5, 1 : 1.0 and 1 : 2.0. This PVP modification allows NPs to disperse uniformly in the composite without agglomeration of NPs. The optimized condition is 1 : 0.5 molar ratio for having narrow particle size distribution in PSA.

#### 3.4.1 TGA and FT-IR analysis after surface modification

**Fig. 11:**
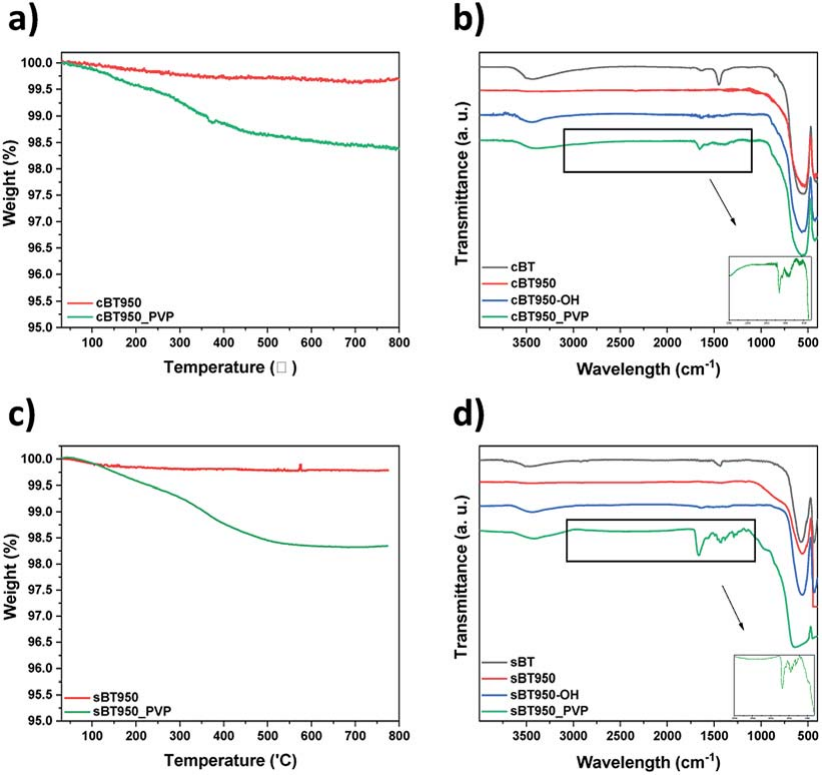
After surface modification of BT (a) TGA data of cBT (b) FT-IR spectrum of cBT, (c) TGA data of sBT and (d) FT-IR spectrum of sBT.

BT agglomerated and sank to the bottom when they mixed with the PDMS elastomer. So, after the hydroxylation of the BT950, the NPs were modified with PVP to increase dispersion in the PDMS elastomer. As shown in Fig. 11, the PVP content and C=O functional group of surface modified BT950 (BT950_PVP) were measured by TGA and FT-IR analysis, respectively. The weight loss in Fig. 11a and c are similar at 350 °C with 1.5 wt%. The FT-IR spectrum of PVP was also identified as shown in Fig. 11b and d; 1652 cm^−1^ (C=O), 1460 cm^−1^ (CH_2_, CH_3_) and 1294 cm^−1^ (CN). The surface of the NPs was also observed before and after surface modification using FE-SEM. The particle size of sBT950_PVP and cBT950_PVP is increased slightly compared to unmodified BT950 as shown in Fig. S7.†

#### 3.4.2 Dielectric property after modification

**Fig. 12:**
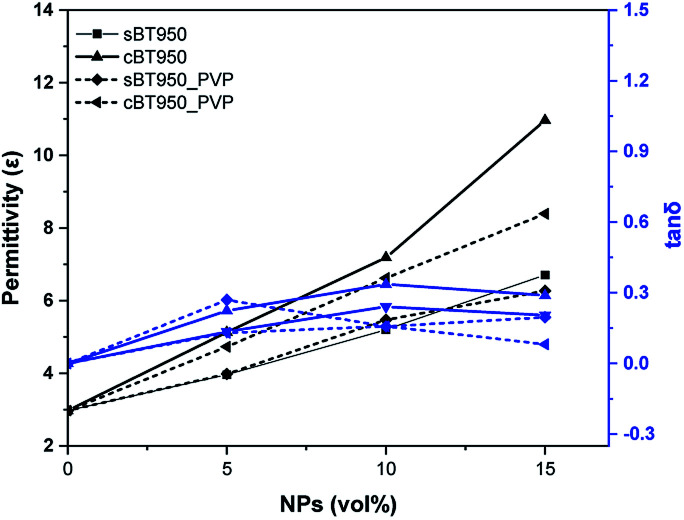
Dielectric properties of BT-PDMS composite before and after PVP modification. Permittivity (*ε*) and tan *δ* at 0.1 MHz.

In addition, the dielectric property was measured after surface modification. The chemical treatment does not result in a noticeable difference in permittivity of the composites without 15 vol% of the cBT950_PVP composite, as shown in Fig. 12. It was measured to be 24% lower than the unmodified cBT950 composite. This suggested that PVP coated on the NPs surface could be acting as an insulating layer between the NPs and PDMS. As the content of PVP increases, the relative quantity of BT950 content is lowered, which might lead to a decreased dielectric constant. Nevertheless, the surface modification of NPs must be treated due to prevent agglomeration and sink. If the NPs are non-uniformly dispersed in matrix, the properties of composite are not be and the agglomerated NPs can be work as defects when fabricating a robotic gripper. The defect in the micro-scale patterned electro-adhesive gripper could bring the electrical breakdown or bad surface roughness. This optimization makes it feasible to apply soft-robotic applications.

#### 3.4.3 Surface hardness after modification

**Fig. 13:**
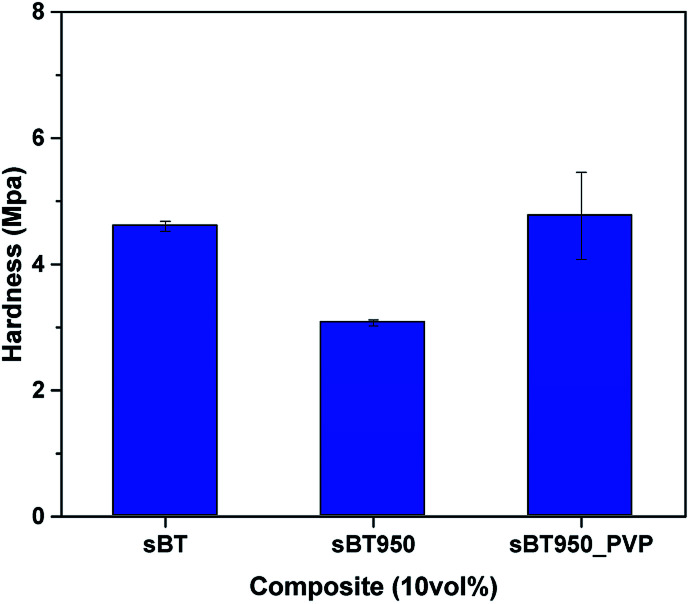
Surface hardness of 10 vol% composite. sBT, sBT950 and sBT950_PVP composites.

In Fig. 13, the surface hardness of the 10 vol% composite was measured by a nano indenter under conditions of strain 0.1 s^−1^ and maximum depth 9 μm. When sBT950 was modified with PVP, it showed that the surface hardness was improved by 55% or more due to the well-dispersed sBT950_PVP. As mentioned above, the surface hardness of the sBT composite was diminished due to the sink of NPs after the phase transition, but it is dramatically increased after the PVP surface modification. The hardness of sBT_PVP is missed due to the fluctuation of experimental results. Similar to this result, the PVP sBT950_PVP showed large error range more than 1 MPa distinct from sBT or sBT950.

#### 3.4.4 BT dispersion in PDMS elastomer

**Fig. 14:**
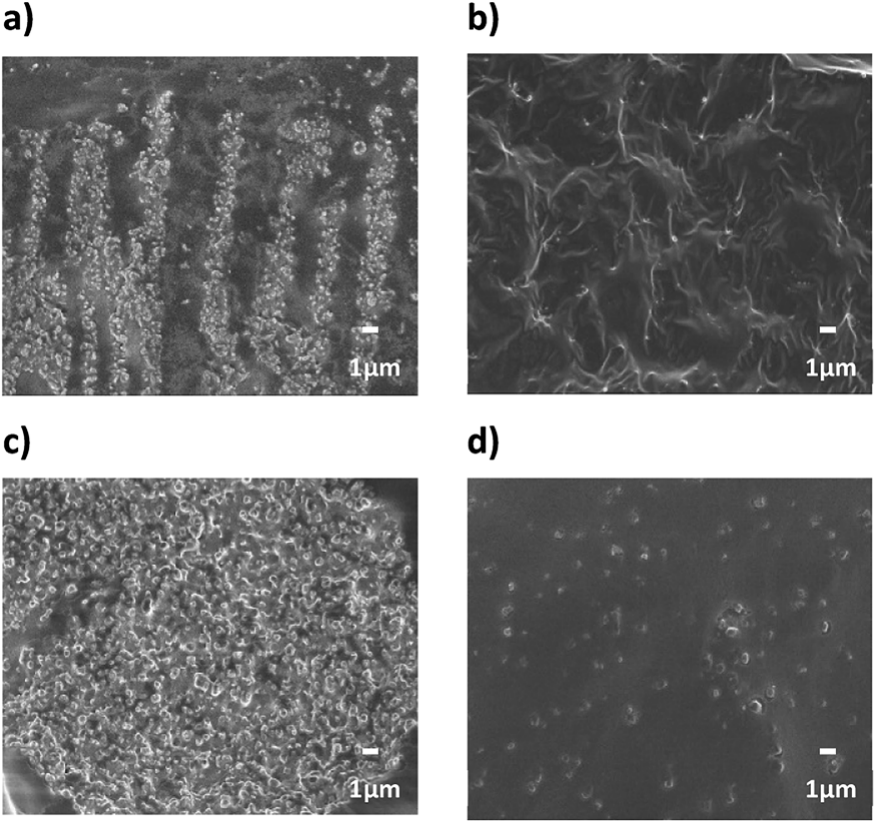
FE-SEM cross-section images of 10 vol% NPs composites (a) cBT950, (b) cBT950_PVP composite, (c) sBT950 and (d) sBT950_PVP composite (×5000).

The dispersion of BT in PDMS elastomer was confirmed by FE-SEM images. In Fig. S8,† the cross-section of the 10 vol% composite was observed at low magnification (×500) to confirm the aggregation and dispersion of the NPs in the PDMS. Before the modification, the BT and BT950 NPs showed large agglomeration even after physical dispersion in PDMS elastomer using a planetary mixer. Depending on the shape, the degree of aggregation was also different. In the sBT NPs composite, the NPs aggregated like an elliptical state, compared to the cBT NPs which were arranged in a long, stretched form like a coral reef. This indicates that the NPs shape affect aggregation and increases the surface area by forming coral-like reef, and may show a difference in permittivity between sBT and cBT. On the other hand, FE-SEM images show that the BT950_PVP NPs are well dispersed in the PDMS elastomer. In Fig. 14, the cross-section at high magnification (×5000) shows the details of the agglomerated morphology. In particular, it is observed that after surface modification, most of the NPs have improved dispersion in the composite. It indicates that PVP surface modification improves the dispersion of NPs in the PDMS elastomer. After physically mixed, the PVP coated NPs well interact with PDMS matrix and it helps to maintain dispersion without sinking. Although the permittivity of the composite is high, if the NPs dispersion in the composite is poor, it affects the composite properties. When a high voltage is applied to the sample, for example, defects would appear or a non-uniform sample would be produced, which results in leakage of current, local heating, and finally, electrical breakdown. Therefore, in addition to permittivity, dispersion is also an important factor for electric devices including dielectric elastomer.

**Fig. 15:**
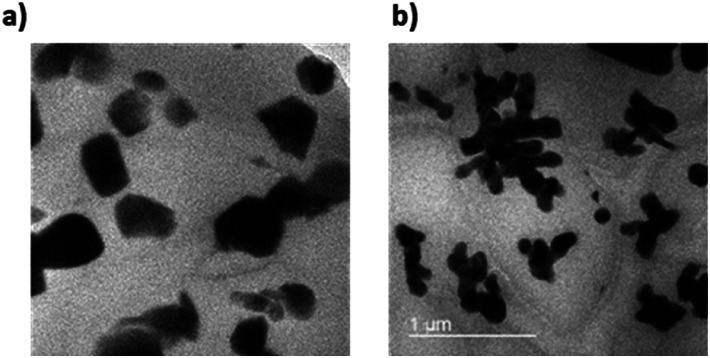
FE-TEM images of 10 vol% NPs composites. (a) sBT950_PVP composite and (b) cBT950_PVP composite.

FE-TEM images were measured to confirm the dispersion of the NPs in composite. As shown in Fig. 15, TEM images showed fine NPs dispersion in PDMS composite regardless of the shape. The size of NPs are also matched the result of PSA in Fig. S5 and S6.† It also demonstrated that the particle shape of coral reef still maintained even after PVP modification.

## 4. Conclusions

We have investigated the effect of cBT on the dielectric properties and dispersion in PDMS elastomer. Compared with the sBT, the PDMS composites with the cBT showed enhanced dielectric properties. In particular, the composites with 15 vol% of the cBT had a 64% higher permittivity than the sBT. This influences the dielectric characteristics depending on the morphology, because the specific surface area of the coral-like shape is approximately 4 times higher than the specific surface area of the spherical shape. Phase transition and surface modification were also employed to increase the permittivity and dispersion of the BT in the PDMS composite. As a result, in 15 vol% composites, the permittivity of cBT950_PVP composite was increased 25.4% to 8.4 higher than the sBT composite, 6.7. Finally, the cBT950_PVP–PDMS composite was applied to obtain a high dielectric elastomer which is flexible and has a uniform NPs dispersion. This could be used in electric devices such as robotic grippers, pyroelectric devices, and piezoelectric devices which need elasticity and high permittivity.

## Conflicts of interest

There are no conflicts to declare.

## Supplementary Material

RA-010-D0RA04196C-s001
