# Supplementary material for: Synthesis of BaTiO3 nanoparticles as shape modified filler for high dielectric constant ceramic–polymer composite
Source: RSC Adv. 2020 Aug 7;10(49):29278–86. doi: 10.1039/d0ra04196c (PMC9055919; doi:10.1039/d0ra04196c)
Supplement: RA-010-D0RA04196C-s001 [file RA-010-D0RA04196C-s001.pdf]

Electronic Supplementary Material (ESI) for RSC Advances.
This journal is © The Royal Society of Chemistry 2020

Supplementary information

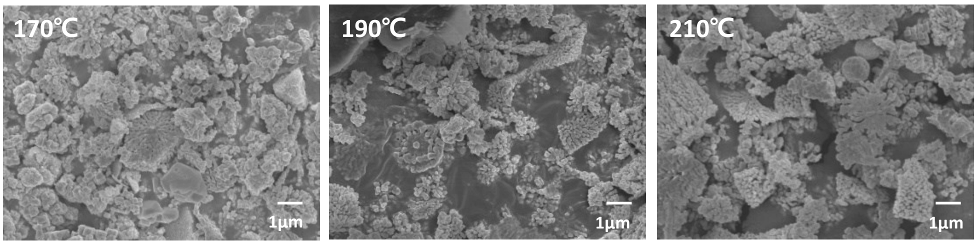

Fig. S1 FE-SEM images of BT particles after hydrothermal synthesis by reaction temperature (x5000)

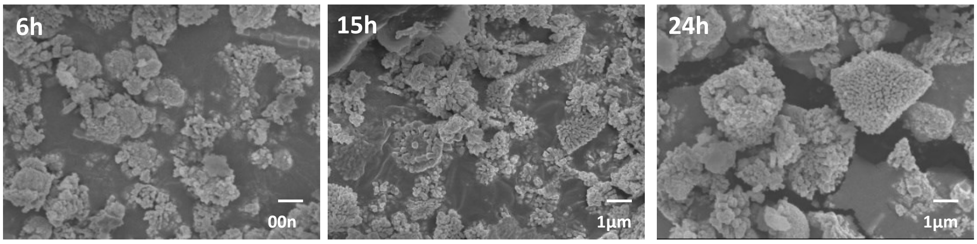

Fig. S2 FE-SEM images of BT particles after hydrothermal synthesis by reaction time (x5000)

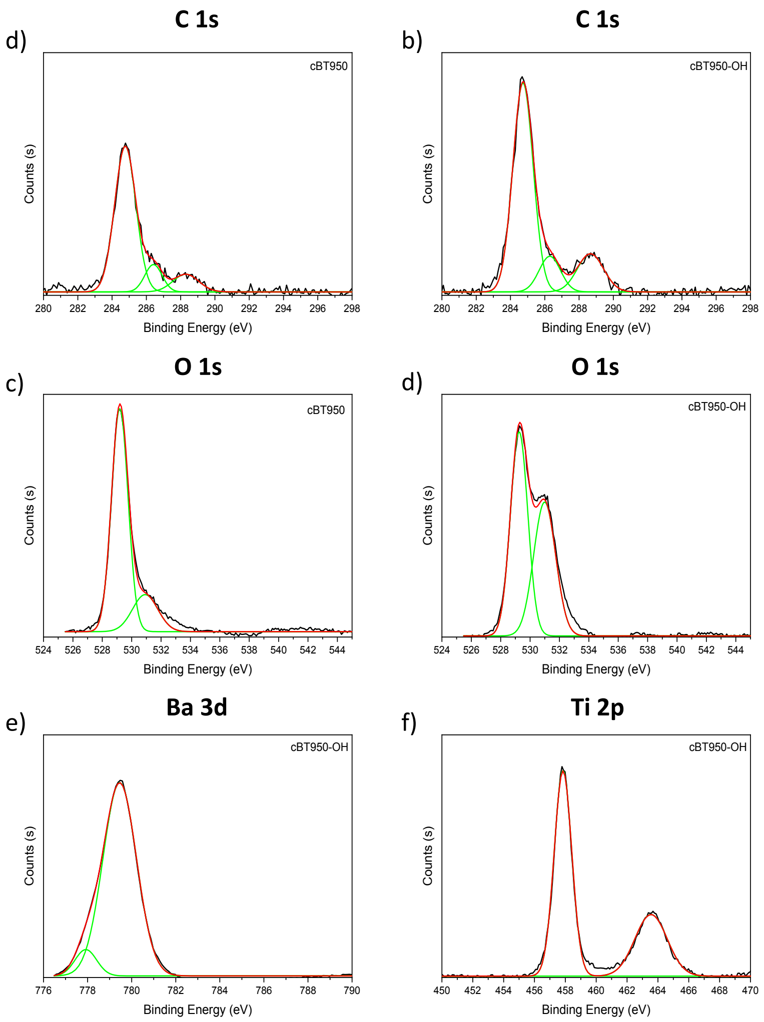

Fig. S3 XPS photoelectron spectra of cBT950 before and after hydroxylation. a) cBT950 C 1s, b) cBT950-OH C 1s, c) cBT950 O 1s, d) cBT950-OH O 1s, e) cBT950-OH Ba 3d and f) cBT950-OH Ti 2p

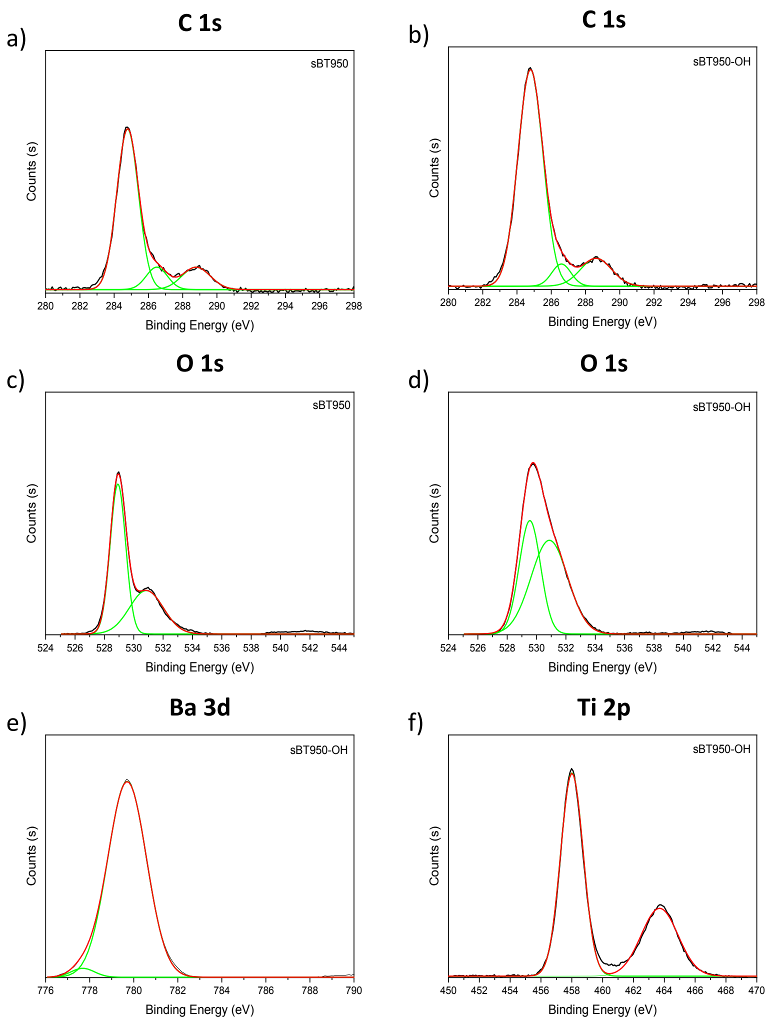

Fig. S4 XPS photoelectron spectra of sBT950 before and after hydroxylation.a) sBT950 C 1s, b) sBT950-OH C 1s, c) sBT950 O 1s, d) sBT950-OH O 1s, e) sBT950-OH Ba 3d and f) sBT950-OH Ti 2p

Table.S1XPS integrated peak area of C 1s, O 1s, Ba 3d and Ti 2p in cBT950 series

	**C 1s**		**O 1s**		**Ba 3d**		**Ti 2p**	
	**BE(eV)**	**Intensity integrated from peak area**	**BE(eV)**	**Intensity integrated from peak area**	**BE(eV)**	**Intensity integrated from peak area**	**BE(eV)**	**Intensity integrated from peak area**
**cBT950**	284.77	6599	529.18	29197				
	286.41	1013	530.36	7418				
	288.30	979						
**cBT950-OH**	284.73	9070	529.26	27432	777.92	92149	457.85	21339
	286.29	1628	530.99	22823	779.46	8047	463.55	11720
	288.67	2141						

Table.S2XPS integrated peak area of C 1s, O 1s, Ba 3d and Ti 2p in sBT950 series

	**C 1s**		**O 1s**		**Ba 3d**		**Ti 2p**	
	**BE(eV)**	**Intensity integrated from peak area**	**BE(eV)**	**Intensity integrated from peak area**	**BE(eV)**	**Intensity integrated from peak area**	**BE(eV)**	**Intensity integrated from peak area**
**sBT950**	284.78	35984	528.92	122435				
	286.59	7040	530.86	77519				
	288.62	4868						
**sBT950-OH**	284.78	58303	529.54	128342	777.69	534974	458.01	123725
	286.49	4542	530.87	182148	779.70	14707	463.69	67286
	288.78	9218						

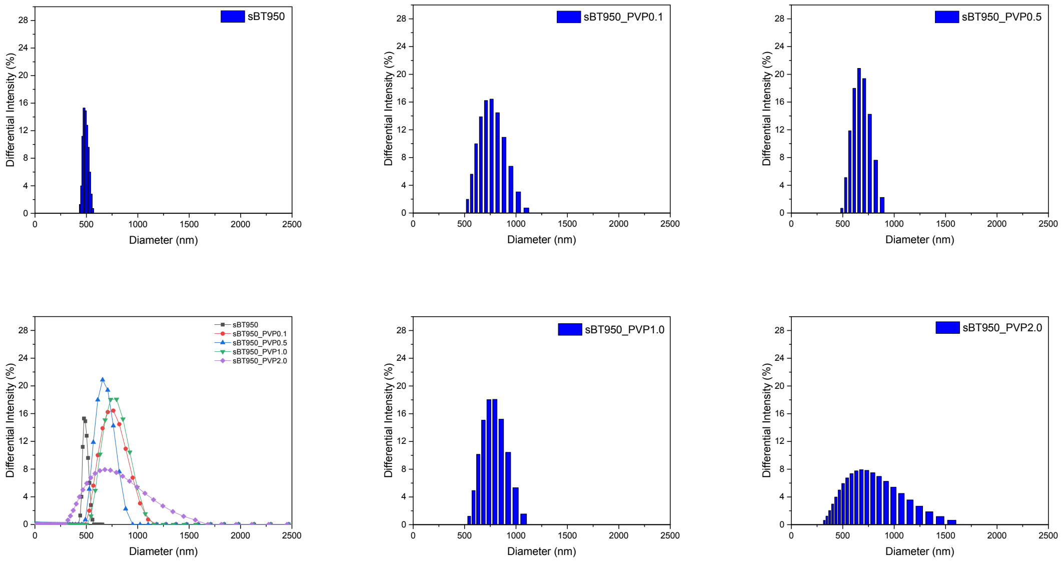

Fig. S5 Particle size distribution of sBT950 series after modification by measuring PSA

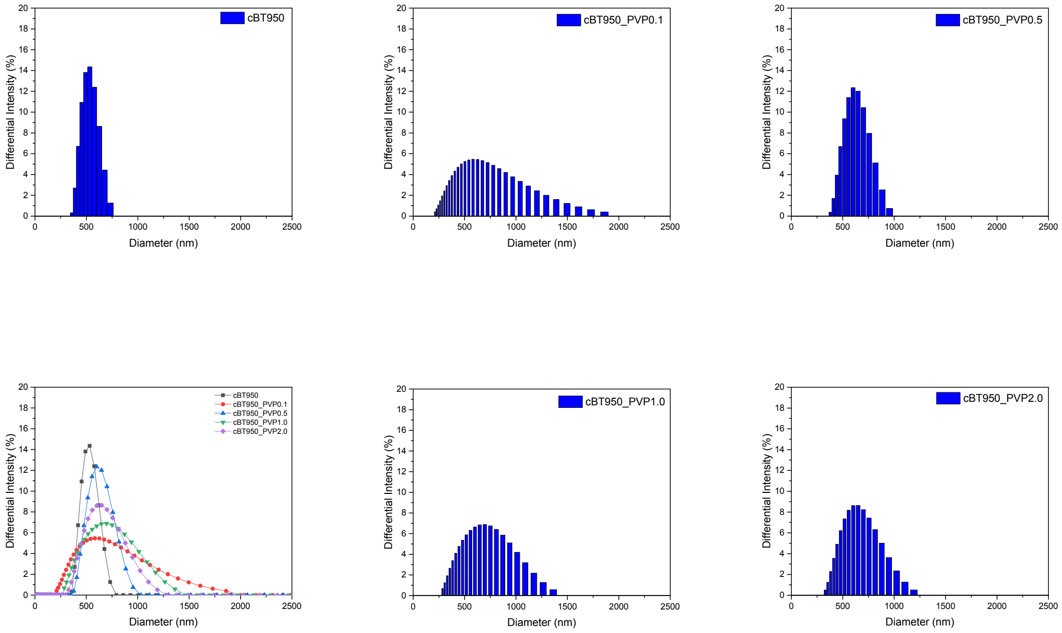

Fig. S6 Particle size distribution of cBT950 series after modification by measuring PSA

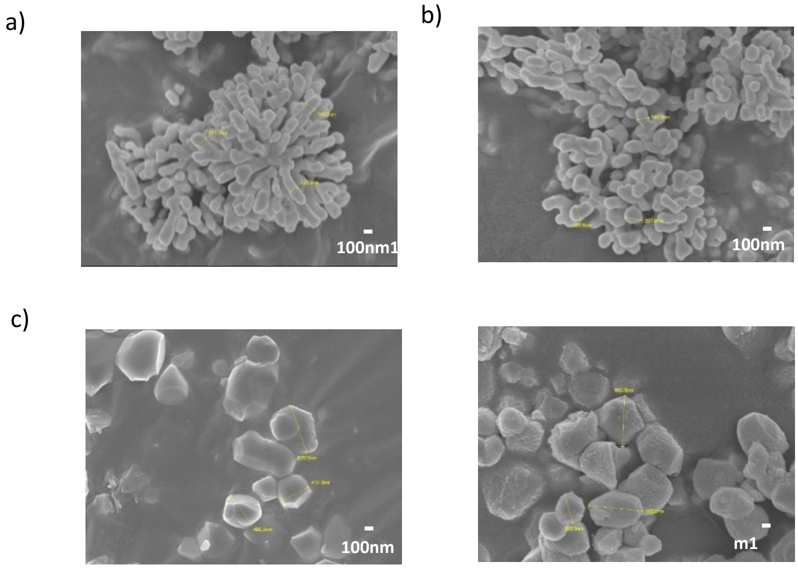

_PVPBT950sBT950 and d) _PVP,c) sBT950cBT950 b) c particle a) $_3$ FE-SEM images of BaTiO**7 S.Fig**

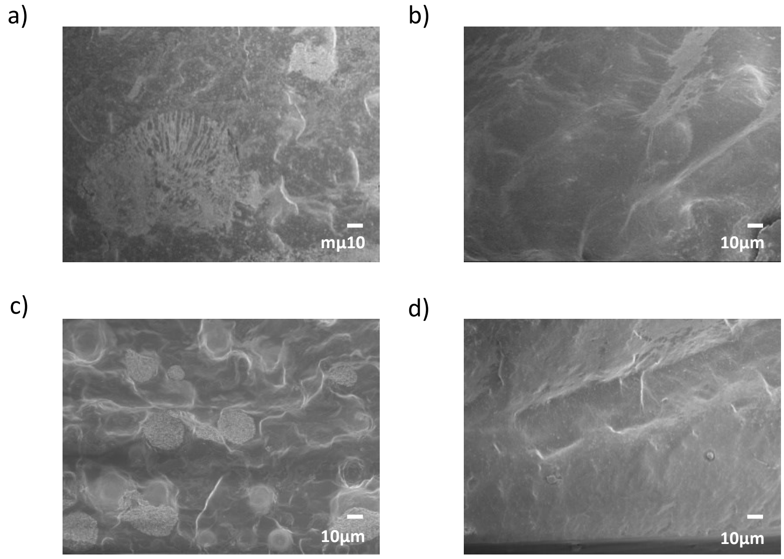

Fig. S8 FE-SEM images of $BaTiO_3$ composite in PDMS. (x500) a) cBT, b) cBT950_PVP, c) sBT and d) sBT950_PVP composite
